# Lapatinib-Loaded Nanocapsules Enhances Antitumoral Effect in Human Bladder Cancer Cell

**DOI:** 10.3389/fonc.2019.00203

**Published:** 2019-04-09

**Authors:** Julieti Huch Buss, Karine Rech Begnini, Franciele Aline Bruinsmann, Taíse Ceolin, Mariana Souza Sonego, Adriana Raffin Pohlmann, Sílvia Stanisçuaski Guterres, Tiago Collares, Fabiana Kömmling Seixas

**Affiliations:** ^1^Molecular and Cellular Oncology Research Group, Laboratory of Cancer Biotechnology, Technology Development Center, Federal University of Pelotas, Pelotas, Brazil; ^2^Pharmaceutical Sciences, Federal University of Rio Grande do Sul, Porto Alegre, Brazil; ^3^Postgraduate Program in Biotechnology, Technology Development Center, Federal University of Pelotas, Pelotas, Brazil; ^4^Institute of Chemistry, Federal University of Rio Grande do Sul, Porto Alegre, Brazil

**Keywords:** bladder cancer, her-positive, epidermal growth factor receptor (EGFR), tyrosine kinase inhibitor, nanocapsules, lapatinib

## Abstract

Transitional cell carcinoma (TCC) represents the most frequent type of bladder cancer. Recently, studies have focused on molecular tumor classifications in order to diagnose tumor subtypes and predict future clinical behavior. Increased expression of HER1 and HER2 receptors in TTC is related to advanced stage tumors. Lapatinib is an important alternative to treat tumors that presents this phenotype due to its ability to inhibit tyrosine kinase residues associated with HER1 and HER2 receptors. This study evaluated the cytotoxicity induced by LAP-loaded nanocapsules (NC-LAP) compared to LAP in HER-positive bladder cancer cell. The cytotoxicity induced by NC-LAP was evaluated through flow cytometry, clonogenic assay and RT-PCR. NC-LAP at 5 μM reduced the cell viability and was able to induce G0/G1 cell cycle arrest with up-regulation of p21. Moreover, NC-LAP treatment presented significantly higher apoptotic rates than untreated cells and cells incubated with drug-unloaded nanocapsules (NC) and an increase in Bax/Bcl-2 ratio was observed in T24 cell line. Furthermore, clonogenic assay demonstrated that NC-LAP treatment eliminated almost all cells with clonogenic capacity. In conclusion, NC-LAP demonstrate antitumoral effect in HER-positive bladder cells by inducing cell cycle arrest and apoptosis exhibiting better effects compared to the non-encapsulated lapatinib. Our work suggests that the LAP loaded in nanoformulations could be a promising approach to treat tumors that presents EGFR overexpression phenotype.

## Introduction

Bladder cancer (BC) is a heterogeneous disease which presents several molecular characteristics associated with different clinical outcomes (1). Urothelial or transitional cell carcinoma (TCC) represent the most frequent type of bladder cancer (2) and are classified into two subtypes tumors depending on the formation pathway: non-muscle invasive bladder cancer (NMIBC) or muscle invasive bladder cancer (MIBC) (3). NMIBC are confined to the mucosa (stage Ta, CIS) or submucosa (stage T1) and are accounted for 60–80% of the diagnosed bladder cancer cases (4). The histological evaluation is the gold standard for the classification of the tumor allowing the implementation of the best possible therapeutic alternative. This strategy also allows the evaluation of the risks of recurrence and progression of the disease (5, 6). However, recent studies have focused on tumor classifications considering their molecular characteristics in order to diagnose tumor subtypes and establish the best possible therapeutic alternative (7, 8).

Molecular analyzes have shown that approximately 75% of transitional cell carcinomas overexpress epidermal growth factor receptors (EGFR) and their level of expression is directly related to advanced stage tumors (9, 10). EGFR receptors consist of a family of four receptors, EGFR (ErbB1/Her1), EGFR2 (ErbB2/Her2), EGFR3 (ErbB3/Her3), and EGFR4 (ErbB4/Her4) (11, 12), which play an important role in the control of cell proliferation and differentiation (13). However, the EGFR signaling pathway has also been associated with tumor progression and development, through the activation of pathways that result in angiogenesis and increased metastatic potential (14, 15).

HER1 and HER2 receptors have shown altered expression in several types of cancers, including bladder cancer (16, 17). EGFR have in common an extracellular binding domain, a transmembrane portion and an intracellular domain of tyrosine kinase (18, 19). Thus, the pharmacological inhibition of the tyrosine kinase intracellular domain represents an important approach in the development of therapies against tumors which overexpress EGFR. Moreover, it is known that tyrosine kinase inhibitors represent an important class of drugs used in oncology (20).

Lapatinib is a dual tyrosine kinase inhibitor, due to its ability to inhibit both HER1 receptors and HER2-associated tyrosine kinase receptors (21). It was approved in 2007 by the American Food and Drug Administration (FDA) for treatment of advanced or metastatic breast cancer (22) and it is considered an important alternative in the therapy of HER-positive tumors. However, lapatinib presents low bioavailability and low solubility in water (23, 24). Therefore, the nanoencapsulation of lapatinib represents an important approach to increase its apparent solubility in water and consequently its therapeutic effects. In this context, several nanocarriers are under development with the aim of improving therapeutic efficacy of antitumor drugs, improving their solubility, enabling to target them in a specific way and releasing the drug in a controlled manner (25, 26). Knowing that *in vitro* studies are efficient systems which allows the rapid evaluation of different patterns of responses, the objective of this study was to evaluate the cytotoxicity induced by Lapatinib-loaded nanocapsules in HER-positive bladder cancer cell.

## Materials and Methods

### Preparation and Physicochemical Characterization of the Formulations

Lapatinib-loaded nanocapsules (NC-LAP) were prepared by interfacial deposition of pre-formed polymer method (27). Briefly, an organic phase (66 mL of acetone and 9 mL ethanol) containing the polymer (PCL, 0.3000 g), sorbitan monostearate (0.1155 g), copaiba oil (0.474 mL) and lapatinib (0.0025 g) was kept under magnetic stirring at 40°C. After complete dissolution of the components, the organic phase was injected into 90 mL of an aqueous phase, containing polysorbate 80 (0.2310 g), under magnetic stirring at room temperature. After 10 min, the solvents were eliminated and the suspension was concentrated under reduced pressure. The final volume was adjusted to 10 mL. Drug-unloaded nanocapsules (NC) were also prepared, omitting the lapatinib in the organic phase. The formulations were characterized as described below. All analyses were performed in triplicate batches (*n* = 3).

### Drug Content and Encapsulation Efficiency

An analytical method for the quantification of lapatinib was validated using high performance liquid chromatography with UV detection (HPLC-UV). The analysis was performed with a Perkin Elmer Series 200 chromatograph with detection at 260 nm and column Phenomenex Lichrosphere® C18 (4.6 × 150 mm, 4 μm). The composition of the mobile phase was 60% ammonium acetate (20 mM, pH 3.3) and 40% acetonitrile, flow rate of 0.8 mL min^−1^ and injection volume of 20 μL. The analytical method was specific, linear in the range of 1–20 μg mL^−1^ (*r* = 0.9987), precise (RSD <2%) and accurate (99.87 ± 2.63%). The drug content in the NC-LAP (200 μL of formulation) was determined by diluting the samples in 5 mL of the mobile phase. The solution was sonicated for 30 min, and then filtered through a 0.45 μm pore size membrane (Millipore, USA) and assayed by HPLC-UV. The Lapatinib encapsulation efficiency was determined after ultrafiltration-centrifugation (Ultrafree-MC 10 kDa, EMD Millipore, Billerica, MA, USA) at 2,688 × g for 10 min. The ultrafiltrate was quantified by HPLC-UV and the encapsulation efficiency (EE) percentage was calculated by the difference between the total and non-encapsulated drug concentrations divided by the total content multiplied by 100.

### Size Distribution, Zeta Potential, and pH Measurements

The particle size and the size distribution were determined by laser diffraction (Mastersizer® 2000, Malvern Instruments, UK) aiming to evaluate the absence of micrometric particles. The sample was added to the equipment sampling apparatus in an amount sufficient to obtain at least 2% obscuration. The particle size was expressed by the volume-weighted mean diameter [D (3, 4)], and by the diameters calculated at percentiles at 10, 50, and 90 [d_0.1_, d_0.5_, and d_0.9_, respectively] of the size distribution curve. The polydispersity values (Span) were determined using (Equation 1):

(1) $$\begin{array}{r} \text{Span}=\text{d}\left(0.9\right)-\text{d}\left(0.1\right)/d\left(0.5\right) \end{array}$$

The mean particle size (z-average diameter), polydispersity index and zeta potential were determined by dynamic light scattering (DLS) at 25°C using a Zetasizer® Nano ZS (Malvern Instruments, UK). After adequate dilution of samples (250×) in purified and filtered water the correlogram was obtained and the z-average diameter and PDI were calculated by the method of Cumulants. The zeta potential values were determined by electrophoretic mobility in the Zetasizer® instrument after diluting the samples in 10 mM NaCl aqueous solution (500×). The pH values were determined using a calibrated potentiometer (DM-22 Digimed, Brazil) via direct measurements of the formulations.

### Cell Culture and Experimental Conditions

This study was performed using human bladder cancer cell line T24 (EGFR-expressing human bladder carcinoma cell line) obtained from Rio de Janeiro Cell Bank (PABCAM, Federal University of Rio de Janeiro, Brazil). The transitional cell carcinoma cells were cultured in Dulbecco's modified Eagle's medium (DMEM) supplemented with 10% fetal bovine serum (FBS), 1% L-glutamine and 1% penicillin/streptomycin at 37°C and 5% CO_2_ in a humidified incubator. All experiments were performed using cells in the logarithmic growth phase and the results were obtained by averaging three independent experiments performed in triplicate for each experiment. The IC_50_ (concentration that inhibits 50% of cell growth) was also calculated using GraphPad Prism 7.0 Software. The drug vehicle DMSO was calculated to never exceed 0.5% per well.

### Viability Assay

The transitional bladder carcinoma cells viability after different conditions of treatments and time was evaluated by flow cytometry using Guava ViaCount Reagent. T24 cell line was plated in 24-well plates at a density of 5 × 10^4^ cells per well. After adherence period, cells were incubated with medium containing non-encapsulated Lapatinib (LAP) at concentrations of 3.12, 6.25, 12.5, and 25 μM; Lapatinib-loaded nanocapsules (NC-LAP) at concentrations of 0.625, 1.25, 2.5, 5, and 10 μM and with the relative volume of these concentrations of drug-unloaded nanocapsules (NC) for 24, 48, and 72 h. After the different incubations times, cells were washed with phosphate buffered saline (PBS; Gibco®, Carlsbad, USA), centrifuged and stained according to the manufacturer's instructions and analyzed using the Muse Cell Analyzer (EMD Millipore Corporation).

### Apoptosis Induction Analysis

The ability of the different treatments to induce apoptosis against bladder carcinoma cells was assessed by flow cytometry using the Muse® Annexin V and Dead Cell Assay kit (EMD Millipore Corporation). For this analysis, T24 cells were plated in 24-well plates at a density of 5 × 10^4^ cells per well. After 24 h of adhesion, cells were incubated with 5 μM of Lapatinib in its free form (LAP), with 5 μM of Lapatinib-loaded nanocapsules (NC-LAP) and with this relative volume of drug-unloaded nanocapsules (NC) for 48 h. After 48 h of treatment, cells were washed with PBS, trypsinized and centrifuged at 1,200 rpm for 10 min. After centrifugation, 1 × 10^5^ cells were stained according to the manufacturer's instructions and analyzed using the Muse Cell Analyzer (EMD Millipore Corporation).

### Cell Cycle Analysis

Cell cycle analysis was performed with the objective of identifying cell populations in different phases of the cell cycle after different treatments. For this analysis, T24 cells were plated in 24-well plates at a density of 5 × 10^4^ cells per well. After 24 h, cells were incubated with 5 μM of Lapatinib (LAP), with 5 μM of Lapatinib-loaded nanocapsules (NC-LAP) and with this relative volume of drug-unloaded nanocapsules (NC) for 48 h. Afterwards, cells were detached, fixed with 70% ethanol and stained according to the manufacturer's protocol. DNA content measurement was analyzed by propidium iodide staining using Guava Cell Cycle reagent kit (Merck Millipore Corporation) and analyzed in Muse Cell Analyzer (EMD Millipore Corporation).

### Analysis of Colony Formation

Clonogenic assay was performed to determine the ability of the different treatments to reduce cell colonies formation. For this assay, T24 cell line was plated in 6-well plates at a density of 2 × 10^3^ cells per well. After 24 h, cells were treated with 5 μM of Lapatinib (LAP), with 5 μM of Lapatinib-loaded nanocapsules (NC-LAP) and with this relative volume of drug-unloaded nanocapsules (NC) for 48 h. After 48 h of treatment, the medium was replaced, and the cells were maintained under controlled atmosphere (37°C with 5% CO_2_ and 95% humidity) for 15 days. After this period, the medium was removed and the cells were washed with PBS, fixed with methanol:acetone (3:1) and stained with crystal violet for 20 min. Subsequently, colonies were diluted in 33% acetic acid and the absorbance of each well was read on a microplate reader at a test wavelength of 595 nm. The perceptual of colony formation was calculated by comparing the means of absorbances obtained from the different treatments and the absorbance values will be proportional to the number of stained cells in the colonies.

### Analysis of Gene Expression

The analyses of gene expression associated with apoptosis induction and cell cycle arrest were investigated by Quantitative Real-Time PCR (qRT-PCR). Cells were added to 24-well plates at a density of 2 × 10^5^ cells per well and grown at 37°C in a humidified atmosphere of 5% CO_2_, 95% air for 24 h. Cells were then treated with 5 μM of Lapatinib (LAP), with 5 μM of Lapatinib-loaded nanocapsules (NC-LAP) and with this relative volume of drug-unloaded nanocapsules (NC) for 8 h. Total RNA isolation, cDNA synthesis, and qRT-PCR were conducted as previously described (28). Briefly, total RNA was isolated using the TRIzol™ Reagent (Invitrogen™, USA). RNA concentration and quality were evaluated using the Nanovue 4282 spectrophotometer (GE Healthcare) and A_260_/A_280_ and A_260_/A_230_ ratios were analyzed. Samples were then digested with DNase by DNA-free kit (Ambion, USA) and cDNA synthesis was performed using the High Capacity cDNA Reverse Transcription Kit (Applied Biosystems, UK) according to the manufacturer's protocol. Quantitative Real-Time PCR reactions were performed on a Stratagene Mx3005P Real-Time PCR System (Agilent Technologies, USA) using SYBR Green PCR Master Mix (Applied Biosystems, UK) and the specific primers described in Table 1. The relative expression data were calculated according to the 2^−ΔΔ*Ct*^ method and were presented as fold changes (29).

**Table 1:** Primers sequences used in this study.

**Gene**	**Sequence 5′-3′**
p21 For	TGTCCGTCAGAACCCATGC
p21 Rev	AAAGTCGAAGTTCCATCGCTC
Bax For	ATGCGTCCACCAAGAAGC
Bax Rev	ACGGCGGCAATCATCCTC
Bcl-2 For	GTGTGGAGAGCGTCAACC
Bcl-2 Rev	CTTCAGAGACAGCCAGGAG
Caspase-3 For	CAGTGGAGGCCGACTTCTTG
Caspase-3 Rev	TGGCACAAAGCGACTGGAT
Caspase-8 For	GGATGGCCACTGTGAATAACTG
Caspase-8 Rev	TCGAGGACATCGCTCTCTCA
Caspase-9 For	CCAGAGATTCGCAAACCAGAGG
Caspase-9 Rev	GAGCACCGACATCACCAAATCC
GAPDH For	GGATTTGGTCGTATTGGG
GAPDH Rev	TCGCTCCTGGAAGATGG

### Data Analysis

Data were expressed as mean ± standard error of the mean (SEM) from three independent experiments performed in triplicate for each experiment. IC_50_ value was determined by non-linear regression analysis in the GraphPad Prism 7.0. Software, data are expressed as mean ± SD. Data set were analyzed using one or two-way analysis of variance (ANOVA) followed by Tukey *post-hoc* test for multiple comparisons and significance level was considered at *P* < 0.05 in all analyses.

## Results

### Lapatinib-Loaded Nanocapsules

**Figure 1:**
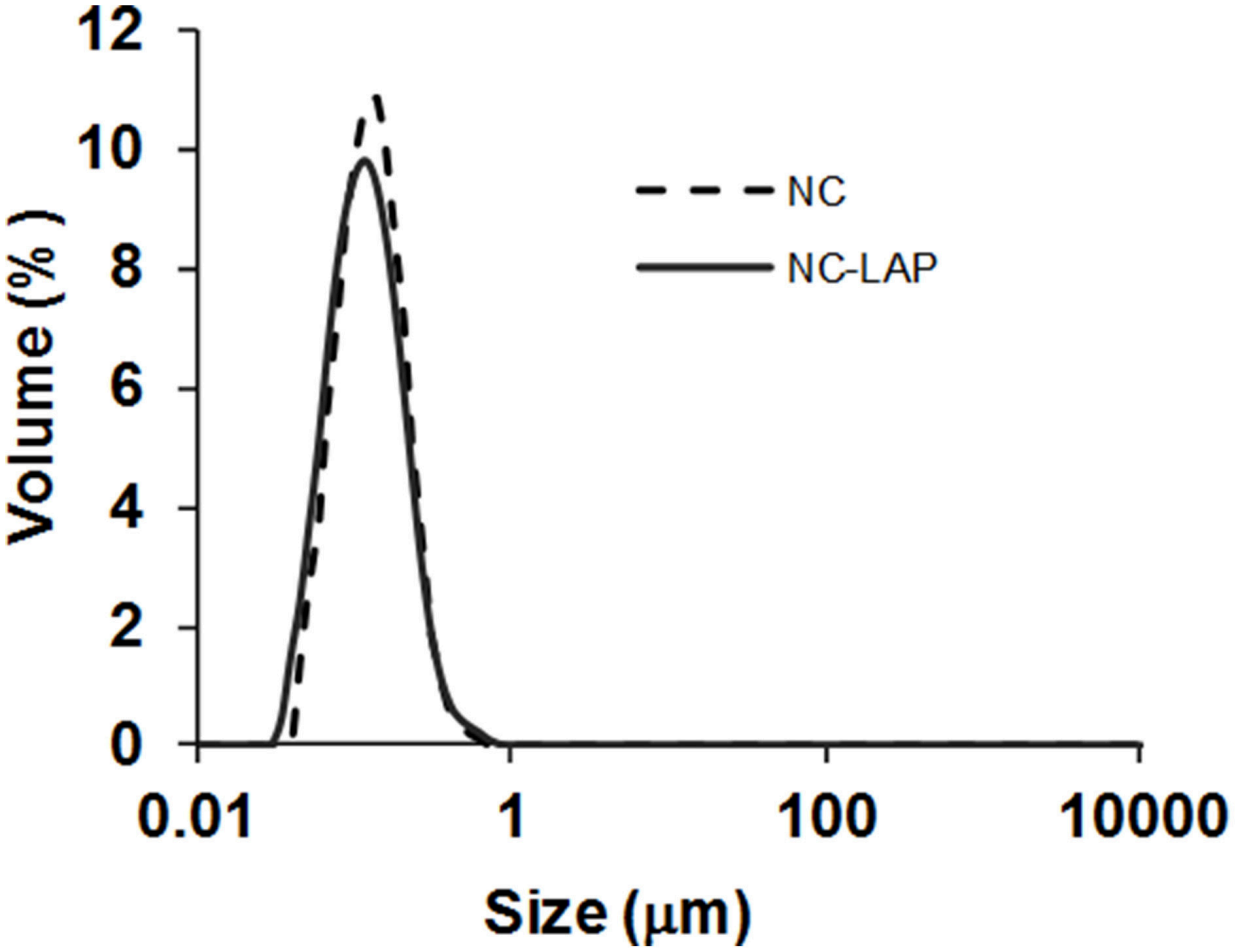
Particle size distribution by volume (laser diffraction): nanocapsules (NC) and lapatinib-loaded nanocapsules (NC-LAP).

Macroscopically, the liquid formulation present an opalescent-white aspect with homogeneous appearance and an odor characteristic of copaiba oil. The total lapatinib content in the NC-LAP was 98.77 ± 2.01% relative to the theoretical value (0.247 ± 0.005 mg mL^−1^), with an encapsulation efficiency of 100%. The formulation containing the drug (NC-LAP) and a control formulation (NC) were analyzed by laser diffraction to determine their particle size distributions. The curves showed unimodal particle size distributions with diameters smaller than 1 μm (Figure 1). Formulations had mean diameters [D (3, 4)] of 148 ± 9 nm (NC) and 146 ± 4 nm (NC-LAP) with polydispersity (Span) of 1.347 ± 0.046 (NC) and 1.406 ± 0.052 (NC-LAP), indicating adequate particle size and narrow size distributions. Since no microscopic contamination was detected by laser diffraction, the formulations were analyzed by dynamic light scattering, electrophorectic mobility and potentiometry. The physico-chemical characteristics (z-average diameter, polydispersity index, zeta potential and pH) are listed in Table 2.

**Table 2:** Results of physicochemical characterization of nanocapsule suspensions.

**Parameter**	**NC**	**NC-LAP**
Z-average diameter (nm)	167 ± 9	172 ± 8
Polydispersity index	0.093 ± 0.010	0.100 ± 0.012
Zeta potential (mV)	−8.28 ± 0.77	−8.85 ± 1.76
pH	5.92 ± 0.10	6.14 ± 0.15

*Results are expressed as mean ± SD*.

The results showed in Table 2 corroborated with the laser diffraction analyses, demonstrating similar particle size, as well as low polydispersity index indicating that the presence of lapatinib in the formulation did not affect theses parameters (*P* > 0.05). In addition, as observed, the formulations of nanocapsules showed low zeta potential values and slightly acid pH values. It is worth mentioning that the mechanism of stabilization for both formulations, NC and NC-LAP, was based on the steric hindrance provided by the polysorbate 80 located at the particle-water interface (30).

### NC-LAP Reduce Viability of Transitional Bladder Carcinoma Cells

**Figure 2:**
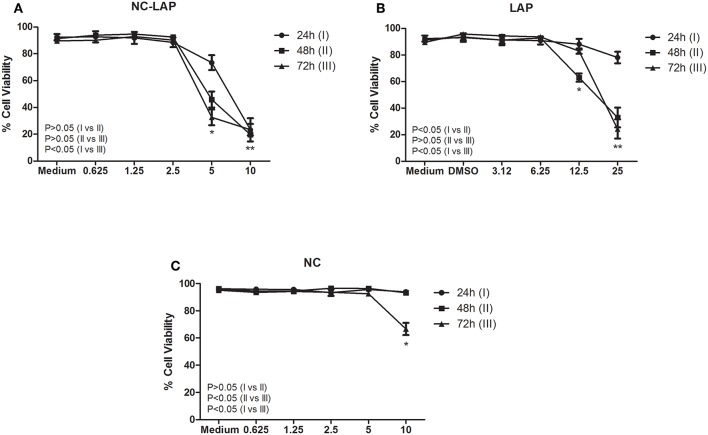
NC-LAP **(A)**, LAP **(B)**, and NC **(C)** effect on T24 cells viability assessed by flow cytometry. Cells were treated with Lapatinib (LAP) at concentrations of 3.12, 6.25, 12.5, and 25 μM, with Lapatinib-loaded nanocapsules (NC-LAP) at concentrations of 0.625, 1.25, 2.5, 5, and 10 μM and with relative volume of these concentrations of blank nanocapsules (NC) for 24, 48, and 72 h. The data are expressed as means ± SEM of three independent experiments. Two-way ANOVA with Tukey *post-hoc* was used to analyze statistical significance. (^*^) represents the significant difference between the different concentrations. The *P*-value represents the significant difference between treatment times. The differences were considered significant at *P* < 0.05.

NC-LAP was able to reduce T24 cells viability showing cytotoxic potential against bladder carcinoma cells (Figure 2A). The results demonstrated that 5 μM of NC-LAP reduced cell viability to 73.39, 45.88, and 32.63% after 24, 48, and 72 h, respectively. Non-encapsulated Lapatinib demonstrated inhibitory capacity at 12.5 and 25 μM after 48 h of treatment, showing viability of 63 and 32.98%, respectively (Figure 2B). Drug-unloaded nanocapsules (NC) showed no cytotoxicity up to 10 μM after 72 h of treatment (Figure 2C). The IC_50_ values, after 24, 48, and 72 h of treatment, for NC-LAP were 6.8 ± 1.2, 5.1 ± 0.6, and 4.5 ± 0.9 μM, respectively, while for LAP in its free form were 17.2 ± 2.8 and 18.7 ± 1.9 μM after 48 and 72 h, respectively. IC_50_ values for NC and for LAP after 24 h were higher than the maximum concentration tested.

### NC-LAP Induces Apoptosis

**Figure 3:**
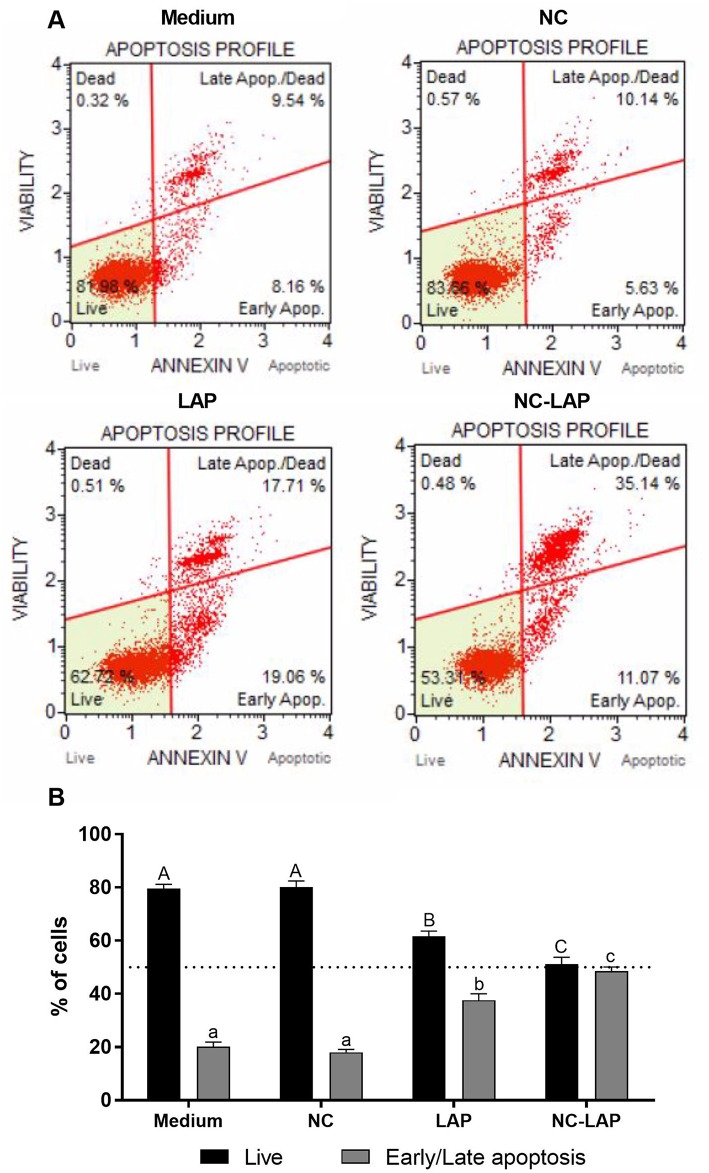
NC-LAP treatment induces apoptosis in T24 cells. T24 cells were incubated with medium (control), LAP or NC-LAP at 5 μM, as well as the relative amount of NC for 48 h. **(A)** Dot Plot shows the percentage of cell live or early/late apoptosis after each treatment determined by Muse® Annexin V and Dead Cell Assay. Dot Plot Upper: Medium (left side) and NC (right side). Dot Plot Bottom: LAP (left side) and NC-LAP (right side). **(B)** The graphic represents the expressed data by mean ± SEM with data from three independent experiments. The one-way ANOVA with Tukey *post-hoc* was used to determine statistical significance. Different capital letters indicate significant differences between means of live cells as well different lowercase letters indicate significant differences between means of cells in early/late apoptosis. Significance was considered at *P* < 0.05.

The rates of early or late apoptosis assessed by flow cytometry showed that NC-LAP at 5 μM induced apoptosis in 48.57% of cells (early or late apoptosis), indicating that NC-LAP is more efficient than solution of free drug in equivalent concentration, which induced 37.65% of the cells (Figure 3). NC treatment was not able to induce apoptosis under analyzed conditions (*P* < 0.05).

### Cell Cycle Arrest Induced by NC-LAP

**Figure 4:**
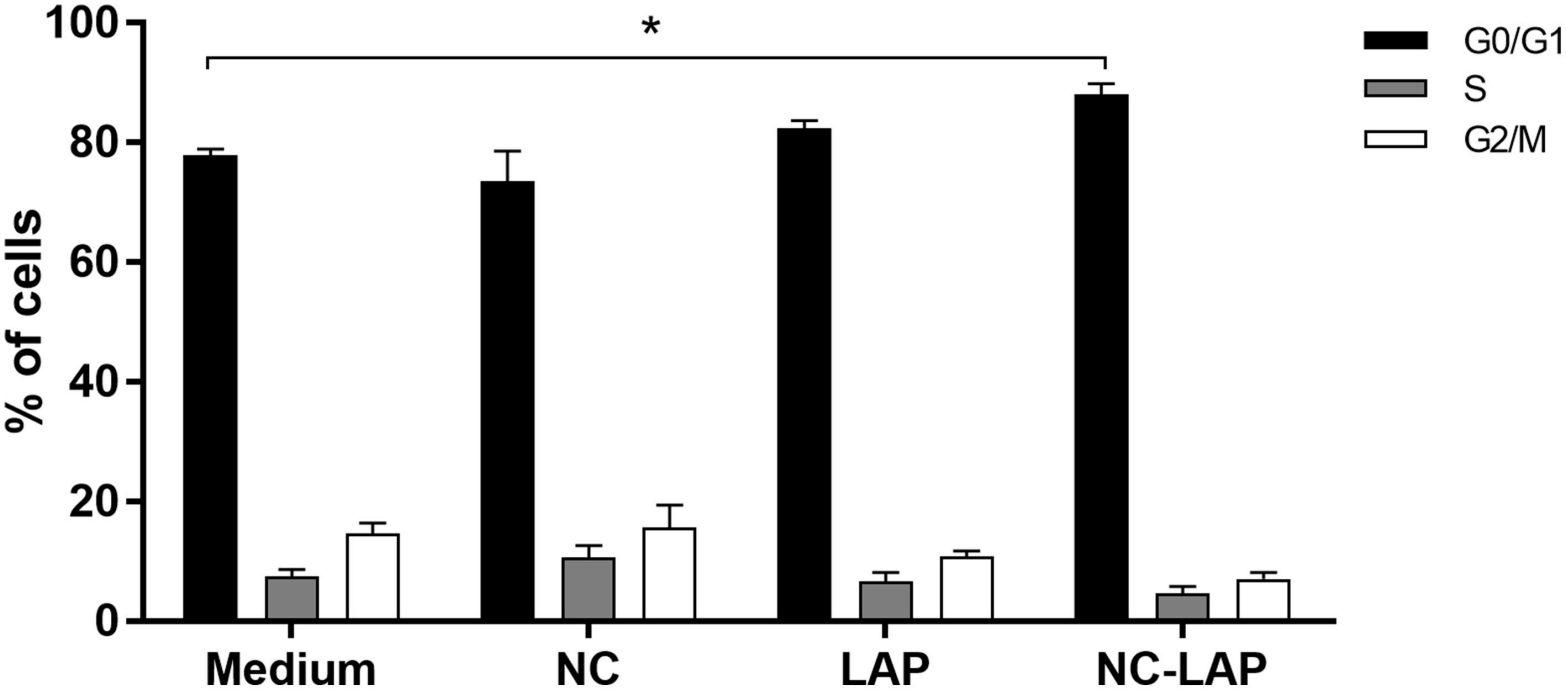
NC-LAP treatment induces cell cycle arrest in T24 cells. T24 cells were treated with LAP or NC-LAP at 5 μM, as well as the relative amount of NC for 48 h. Cell cycle arrest analysis was assessed with propidium iodide staining and flow cytometry. Data are represented by the mean ± SEM with data from three independent experiments. Two-way ANOVA with Tukey *post-hoc* was used to analyze statistical significance. (^*^) represents significant difference (for each phase) between the different treatment in relation to the control group. Significance was considered at *P* < 0.05.

The percentage of cells at the different phases of the cell cycle (G0/G1, S, and G2/M) was analyzed by flow cytometry and it is demonstrated in Figure 4. The results showed that only treatment with NC-LAP at 5 μM for 48 h was able to induce G0/G1 cell cycle arrest in T24 cells when compared to the control group. On the other hand, there were no statistical differences in the S and G2/M phases between tested groups.

### NC-LAP Reduces Colony Formation in Transitional Bladder Carcinoma

**Figure 5:**
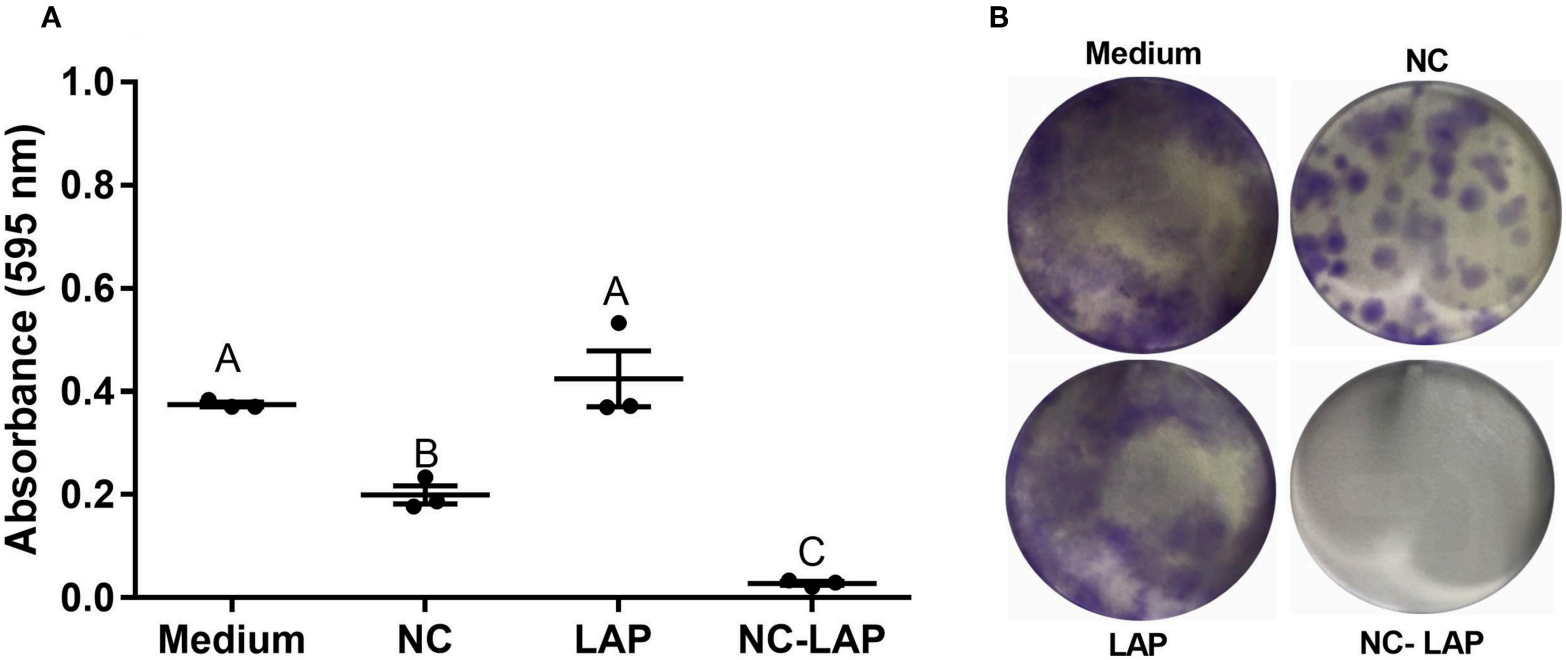
NC-LAP inhibited the T24 cells colony formation. Cells were treated with LAP or NC-LAP at 5 μM, as well as the relative amount of NC for 48 h. **(A)** Shows absorbance relative to each well and **(B)** shows photographs of representative clonogenic assay plates. Data are represented by the mean ± SEM from three independent experiments. One-way ANOVA with Tukey *post-hoc* was used to analyze statistical significance. Different letters indicate significant differences between the means. Significance was considered at *P* < 0.05.

The analysis of colony formation was assessed after treatment with NC-LAP or LAP, as well as the amounts of NC for 48 h. The results showed that NC and NC-LAP at 5 μM have antitumoral potential, reducing T24 colony formation when compared to the control group. However, NC-LAP treatment was able to reduce on average 12.3 × the absorbance rate when compared to control group (Figure 5). LAP treatment showed no reduction in colony formation (*P* > 0.05).

### NC-LAP Changes Apoptosis and Cell Cycle Genes Expression Levels

**Figure 6:**
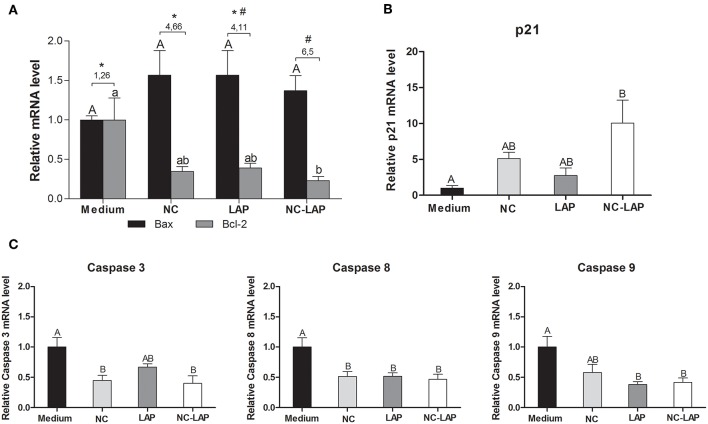
NC-LAP increased Bax/Bcl-2 ratio and led to up-regulation of p21 in T-24 cell line after 6 h of treatment. The gene expression profile was determined by qRT-PCR and data were normalized using GAPDH levels. **(A)** Proapoptotic (Bax) and Antiapoptotic (Bcl-2) gene expression. **(B)** p21 expression gene. **(C)** Expression levels of caspase 3, 8, and 9 after 6 h. Data are represented by the means ± SEM from three independent experiments. The one-way ANOVA with Tukey *post-hoc* was used to analyze statistical significance. Significance was considered at *P* < 0.05. Significance was considered at *P* < 0.05. Different letters indicate significant differences between the means and different symbols (^*^ and #, asterisk and sharp, respectively) indicates difference in the Bax-Bcl-2 ratio levels between groups.

The relative mRNA expression of p21, BAX, and Bcl-2 genes was assessed by qRT-PCR. As demonstrated in Figure 6A, none of the treatments change BAX expression levels in T24 cells. However, Bcl-2 levels were 4.5-fold decreased after treatment with NC-LAP at 5 μM when compared to the control group. Interestingly, Bax/Bcl-2 ratio increased in T24 cells after 5 μM of NC-LAP treatment compared to that observed in untreated cells and LAP and NC treated cells. No effect in mRNA expression levels was observed after NC treatment (*P* > 0.05). Although NC-LAP treatment increased Bax/Bcl-2 ratio and the percentage of cells in early or late apoptosis, there was no increase in expression levels of caspase 3, 8, and 9 (Figure 6C).

The expression level of p21 gene is shown in the Figure 6B. NC-LAP treatment was able to change gene expression profile showing a higher fold induction (10x-fold) when compared to the control group. LAP and NC at 5 μM showed similar fold induction when compared to the control group and treatment with NC-LAP (*P* > 0.05).

## Discussion

Nanotechnology tools have been widely used as drug delivery systems in cancer therapy research (31, 32). These tools have proven to be effective for cancer therapy through controlled drug delivery at specific sites, providing higher intratumoral concentration of chemotherapeutics (33). It has also been shown by our group that nanotechnology-based drug delivery systems could be an important alternative to overcome the resistance developed by cancer cells to drugs (34, 35).

Lapatinib (LAP) is an intracellular inhibitor of tyrosine kinase. Therefore, for its pharmacological action it is crucial that lapatinib is internalized by tumor cells. LAP carried in nanocapsules ensures this increase cellular uptake through the capacity of internalization of the nanocapsules by endocytosis process of the cells (36). In addition, lapatinib-loaded nanocapsules enable an improvement in its oral bioavailability and aqueous solubility and could minimize the ability of LAP to bind albumin or alpha-1 glycoprotein in the blood (37). Herein, we synthesized lapatinib-loaded nanocapsules (NC-LAP) in order to evaluated its cytotoxic activity against HER-positive bladder cancer cells.

The NC-LAP formulation prepared presented appropriate size distributions, indicating a high homogeneity of the particle size (172 ± 8 nm). Nanoparticles with this range of size have enhanced permeability and retention effect (EPR) for drug accumulation in tumors and induced a more efficient therapeutic effect (38, 39). The low zeta potential values observed in our formulation is due to the non-ionic character of polysorbate 80 used in the aqueous phase of the nanocapsule suspensions. Nanocapsule suspensions also showed slightly acid pH values, as expected for formulations containing poly (ε-caprolactone) and copaiba oil (40). Studies have shown that polymer nanomaterials may exhibit more than just inert functions and can alter the expression profile of selected genes and drastically alter cellular responses to these agents (41–43). Thus, it is expected that drug-free nanoformulations have minimal antitumor effect, especially in long term treatment, as observed in the clonogenic assay. The physicochemical characterization of the formulations is a crucial step aiming to ensure that nanotechnological properties are achieved (44).

In this study, encapsulation of LAP resulted in a statistically significant increase of lapatinib cytotoxicity against T24 bladder cancer cells. Treatment with 5 μM of NC-LAP significantly reduced T24 cells viability and similar results were obtained only with 25 μM of LAP. We also demonstrate here that the reduction in the percentage of viable cells remains after 72 h of treatment, suggesting the sustained release of the drug in the nanoformulation. It is worth mentioning that drug-unloaded nanocapsules did not show any cytotoxic effect at these concentrations in our study. More than that, through clonogenic assay we demonstrate that NC-LAP treatment completely inhibited the ability of cells to form new colonies. Clonogenic assay identifies cells that maintain their reproductive capacity (45) and is an important method to determine cell reproductive capability reestablishment after treatment with cytotoxic agents (46). In this work, almost all cells with clonogenic capacity were eliminated after NC-LAP treatment which was not true for non-encapsulated LAP treatment.

Studies have shown that lapatinib is able of strongly inhibit cell proliferation and induce cell cycle G1 arrest and apoptosis in bladder cancer cells (47). Here we also demonstrate that NC-LAP was able to induce the T24 cell to apoptosis. The apoptotic process can be activated through death receptors or via mitochondria, characterizing the intrinsic or extrinsic pathway for caspase activation (47). In the present study, treatment with NC-LAP had no effect on the expression levels of caspases measured by RT-PCR. On the other hand, NC-LAP treatment resulted in increasing of Bax/Bcl-2 mRNA expression, suggesting that the apoptotic process may be occurring through mitochondria activation, which is controlled by the balance and interactions between members of the Bcl-2 family proteins (48). It has been proposed that the ratio between Bcl-2 and Bax genes seems to be crucial to determine the fate of the cell and an increase of Bax/Bcl-2 ratio results in loss of mitochondrial membrane potential and consequently cell death (49).

In this work, NC-LAP also led to cell cycle arrest in G0/G1 phase, which was not observed for non-encapsulated LAP. RT-PCR analysis showed that NC-LAP treatment also up-regulated p21 expression levels. The cell cycle progression is a process regulated by the activity of cyclin-dependent kinases (Cdk) (50). The p21 protein inhibits the cyclin-dependent kinases pathways regulating negatively the cell cycle progression (51). Cycle arrest at G0/G1 transition in human bladder cancer cells may have been due to the enhanced of expression of p21 with a decrease in cyclin E1, CDK2, and CDK4 kinase levels (52). Our data also suggest that cell viability reduces after NC-LAP treatment due to accumulation of cells in the G0/G1 phase of the cell cycle and induction of apoptosis. In addition, after NC-LAP treatment, it was possible to observe an almost complete inhibition of cells with reproductive potential. Our data agrees with the literature, since it has already been reported that lapatinib strongly inhibited cell proliferation and induced cell cycle G1 arrest and apoptosis in bladder cancer cells (17, 47).

In conclusion, we demonstrate that Lapatinib-loaded nanocapsules showed cytotoxic effect against HER-positive bladder cancer cell. NC-LAP reduced the viability of T24 bladder cells, inducing G0/G1 cell cycle arrest through up regulation of p21; reducing colony formation and leading cells to apoptosis with increase of Bax/Bcl-2 expression. However, further studies are necessary to understand the pharmacokinetic and toxicological effects of NC-LAP formulation.

## Author Contributions

JHB and KRB conceived, designed, and performed the experiments. FAB, TaC, ARP, and SSG contributed with reagents, materials, analysis tools. JHB, KRB, MSS, TiC, FKS, ARP, and SSG contributed to the analysis of the data. JHB, KRB, and FAB wrote the manuscript. KRB, TiC, FKS, ARP, and SSG were responsible for manuscript review and provide critical intellectual input.

### Conflict of Interest Statement

The authors declare that the research was conducted in the absence of any commercial or financial relationships that could be construed as a potential conflict of interest.
